# Effect of family relatedness on characteristics of estimated IBD probabilities in relation to precision of QTL estimates

**DOI:** 10.1186/1471-2156-11-85

**Published:** 2010-09-26

**Authors:** Gertraude Freyer, Jules Hernández-Sánchez, Natascha Vukasinovic

**Affiliations:** 1 Leibniz Institute for Farm Animal Biology (FBN), D-18196 Dummerstorf, Germany; 2 Institute for Evolutionary Biology, University of Edinburgh, Edinburgh EH9 3JT, UK; 3 IRTA Animal Genetics, 25198 Lleida, Spain; 4 Newsham Choice Genetics, STL Research Center, Chesterfield, MO-63005, USA

## Abstract

**Background:**

A random QTL effects model uses a function of probabilities that two alleles in the same or in different animals at a particular genomic position are identical by descent (IBD). Estimates of such IBD probabilities and therefore, modeling and estimating QTL variances, depend on marker polymorphism, strength of linkage and linkage disequilibrium of markers and QTL, and the relatedness of animals in the pedigree. The effect of relatedness of animals in a pedigree on IBD probabilities and their characteristics was examined in a simulation study.

**Results:**

The study based on nine multi-generational family structures, similar to a pedigree structure of a real dairy population, distinguished by an increased level of inbreeding from zero to 28% across the studied population. Highest inbreeding level in the pedigree, connected with highest relatedness, was accompanied by highest IBD probabilities of two alleles at the same locus, and by lower relative variation coefficients. Profiles of correlation coefficients of IBD probabilities along the marked chromosomal segment with those at the true QTL position were steepest when the inbreeding coefficient in the pedigree was highest. Precision of estimated QTL location increased with increasing inbreeding and pedigree relatedness. A method to assess the optimum level of inbreeding for QTL detection is proposed, depending on population parameters.

**Conclusions:**

An increased overall relationship in a QTL mapping design has positive effects on precision of QTL position estimates. But the relationship of inbreeding level and the capacity for QTL detection depending on the recombination rate of QTL and adjacent informative marker is not linear.

## Background

Studies on quantitative trait loci (**QTL**) in dairy cattle are performed almost exclusively on data from commercial populations. Setting up experimental populations is highly expensive and time consuming. Therefore, the simplest and most popular design for QTL mapping in dairy cattle was the granddaughter design (**GDD**, [1]). Single grandsires establish their "own families" with a number of sons (sires) genotyped for a marker panel, involving phenotypic information on the quantitative trait, based on several hundreds of cows ((grand)daughters).

The methodology to detect QTL in general pedigrees exploiting polymorphism of genetic markers was proposed by Fernando et al. (1989), based on a model where both the allelic QTL effects and the polygenic component are assumed to be random normal deviates [2]. The covariance between individuals for a putative QTL is modeled by the probabilities of sharing alleles identical by descent (**IBD**), based on linked marker genotypes. Such IBD scores are important prerequisites in a two-step procedure to compute variance components using ASREML [3,4]. The major advantage of the variance components approach is the ability to account for relationships among individuals in different families. Pong-Wong et al. (2001) proposed a fast deterministic approach to estimate IBD probabilities by combining the methods of Wang et al. (1995) and Knott and Haley (1998) [5-7]. Consequently, managing inbreeding loops with minimal information loss became feasible. This is of interest, since pedigree patterns harbouring inbred individuals do occur in many animal species, even if inbreeding should be avoided under commercial breeding conditions.

A recent, to our knowledge the first, study has shown that using inbred sires in a pedigree positively exerts QTL detection [8]. However, how this applies is not straightforward, as there was neither different phenotypic variance, nor different (poly)genetic variance between the family structures in the simulation study cited. Sensitivity to environmental changes increases in inbred individuals due to loss of heterozygosity accompanied by impaired ability to react to changing or sub-optimal environmental conditions [10]. Most likely, such inbreeding-specific environmental effects do occur in dairy cattle as well. Moreover, respecting negative inbreeding effects on fertility, health and also on economically important traits would be relevant [9]. But the molecular genetic basis of the inbreeding depression phenomenon is still being examined in model species [11]. Therefore, a realistic basis to include these effects in a simulation model is missing.

The only source of inbreeding effects on QTL position estimates in the recent study was the IBD probability. Investigating IBD parameters in this sense remained an open task. The objective of the simulation study summarized in this paper was to provide more insight into the characteristics of IBD probability in relation to the inbreeding level. Therefore, IBD parameters were examined in pedigrees with four generations and stepwise increasing inbreeding level and slightly varying marker panels for QTL mapping. Moreover, an attempt was made to target the theoretical "optimum inbreeding level" for QTL mapping. An extremely high inbreeding level was necessarily considered in order to evaluate the resulting IBD parameters regarding the theoretical optimum inbreeding level.

## Results and discussion

**Figure 1:**
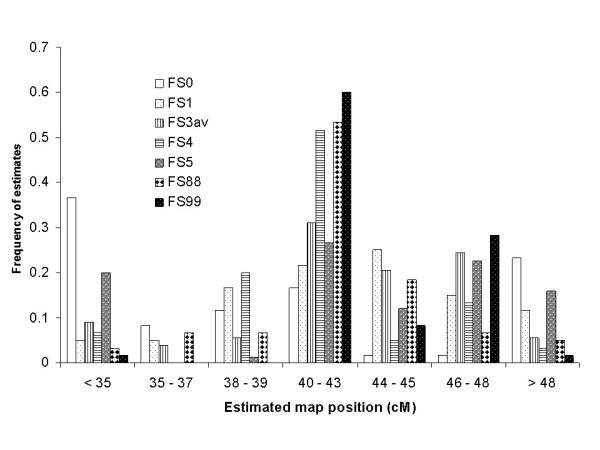
**Frequency of estimated QTL positions (y-axis) over all analyses of single family structures**. FS0, FS1, FS3av (average of FS3, FS3a and FS3b), FS4, FS5, FS88 and FS99, shown in intervals on the chromosomal segment (in cM on the x-axis), where the true QTL position was at 41.5 cM.

Detailed QTL estimates obtained from "mildly inbred" family structures (average F_x _in sires of final offspring increasing from 0 up to 0.042) were reported earlier [8]. The results obtained from the new family structures with higher inbreeding levels followed the trend of positive inbreeding effects on estimated QTL positions, except for FS5 (Figure 1). IBD probabilities deviating along the marked chromosome were mainly responsible for the goodness of QTL position estimates.

Simulation parameters in terms of marker map, number of marker alleles and information content did not affect IBD probability significantly (P-values ranged from 0.75 to 0.87). Family structure, characterized by its inbreeding level, had a significant effect on the IBD probability (P < 0.0001). The IBD parameters were contemplated more detailed in order to identify the reasons for more successfully estimated QTL positions obtained by analysing stronger inbred family structures.

### Means and standard deviations of IBD probabilities

The mean IBD probability (at the true QTL position over all simulation parameters) increased slightly from FS0 to FS88. It was highest in FS99 (Table 1). A minor gap in the general trend was caused by a zero relationship coefficient between the descendants of GGS1 and GGS2 in FS5 (Table 2). All other family structures were linked in both sub-pedigrees. Apart from FS5, ancestral IBD sharing probability of great grandsires GGS1 and GGS2 and their offspring was greater for GGS1 (0.055 in FS0, 0.316 in FS99) than for GGS2 (0.034 in FS0, 0.135 in FS99). The highest IBD sharing probability was in FS99, with both great grandsires equally related to the final offspring by the other great grandsire (see Table 2 for relationship coefficients).

**Table 1:** Mean ± standard deviation of IBD probability for all family structures at 0 cM and at the true QTL position for marker maps M1, M2, M3 and M4 and six marker alleles

	M1	M2	M3	M4
**at 0 cM being the most distant position to the true QTL position:**				
FS0	0.0153 ± 0.0099	0.0155 ± 0.00994	0.0153 ± 0.00987	0.0133 ± 0.0099
FS1	0.0175 ± 0.0116	0.0176 ± 0.0116	0.0175 ± 0.0116	0.0172 ± 0.0116
FS3	0.0192 ± 0.0124	0.0193 ± 0.0116	0.0192 ± 0.0124	0.0187 ± 0.0124
FS3a	0.0223 ± 0.0164	0.0224 ± 0.0166	0.0198 ± 0.0126	0.0206 ± 0.0133
FS3b	0.0205 ± 0.0128	0.0140 ± 0.0112	0.0152 ± 0.0113	0.0252 ± 0.0165
FS4	0.0198 ± 0.0133	0.0212 ± 0.0139	0.0208 ± 0.0135	0.0227 ± 0.0154
FS5	0.0185 ± 0.0136	0.0196 ± 0.0132	0.0181 ± 0.0129	0.0188 ± 0.0089
FS88	0.0407 ± 0.0283	0.0367 ± 0.0220	0.0328 ± 0.0186	0.0379 ± 0.0230
FS99	0.30261 ± 0.0648	0.2785 ± 0.0768	0.3485 ± 0.1015	0.3081 ± 0.1154
**at the true QTL position:**				
FS0	0.0128 ± 0.0106	0.0127 ± 0.0095	0.0127 ± 0.0104	0.0127 ± 0.0104
FS1	0.0170 ± 0.0127	0.0176 ± 0.0127	0.0170 ± 0.0122	0.0170 ± 0.0122
FS3	0.0192 ± 0.0136	0.0191 ± 0.0122	0.0189 ± 0.0132	0.0189 ± 0.0132
FS3a	0.0223 ± 0.0177	0.0224 ± 0.0174	0.0191 ± 0.0169	0.0182 ± 0.0140
FS3b	0.0222 ± 0.0156	0.0142 ± 0.0101	0.0149 ± 0.0126	0.0210 ± 0.0142
FS4	0.0218 ± 0.0159	0.0219 ± 0.0155	0.0200 ± 0.0165	0.0234 ± 0.0151
FS5	0.0214 ± 0.0154	0.0218 ± 0.0137	0.0213 ± 0.0143	0.0179 ± 0.0137
FS88	0.0414 ± 0.0277	0.0369 ± 0.0234	0.0287 ± 0.0227	0.0410 ± 0.0286
FS99	0.2881 ± 0.0919	0.2853 ± 0.0884	0.3392 ± 0.0993	0.3256 ± 0.0994

**Table 2:** Average relationship coefficient among various individuals in the pedigree for different family structures

	average relationship coefficient among animals					
	
Family structure	all	all sires and offspring	GGS1 and his offspring	GGS2 and his offspring	GGS1 and offspring of GGS2	GGS2 and offspring of GGS1
FS0	0.028	0.014	0.189	0.125	0.095	0.051
FS1	0.030	0.015	0.189	0.142	0.101	0.076
FS3	0.031	0.015	0.215	0.115	0.128	0.050
FS3a	0.036	0.026	0.171	0.131	0.114	0.061
FS3b	0.031	0.018	0.196	0.113	0.132	0.055
FS4	0.036	0.018	0.215	0.162	0.128	0.078
FS5	0.034	0.018	0.303	0.275	0.000	0.000
FS88	0.063	0.040	0.258	0.157	0.189	0.103
FS99	0.591	0.254	0.581	0.330	0.581	0.330

### IBD parameters and profiles

**Figure 2:**
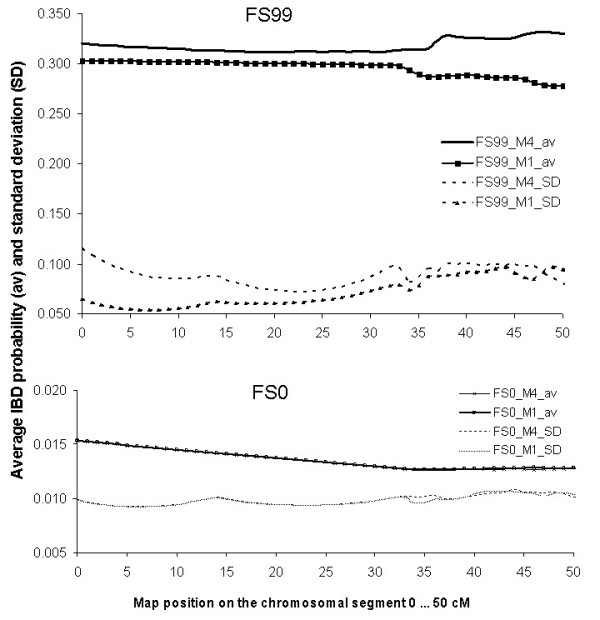
**IBD profiles of averages (av) and standard deviations (SD) at the current map position along the chromosomal segment (0 to 55 cM on the x-axis)**. For family structures FS0 and FS99 in combination with marker maps M1 and M4 and six marker alleles each.

The shape of profiles of IBD parameters along the marked chromosomal region is an indication for the precision of QTL mapping [12]. However, this statement by Grapes et al. (2006) was neither a conclusion from analyzing inbred pedigree structures, nor a result of analyzing practice-like mapping designs. Therefore, we investigated first the profiles of means and standard deviation of IBD along the marker maps (Figure 2). The profiles of average IBD probabilities were flat in almost all family structures. However, FS99 showed a clear break in the course between 34 and 38 cM, as shown in combinations with M1 and M4. This suggests that a recombination happened in the basic generation could be more precisely detected when it was followed by strong inbreeding and manifested in homozygous blocks. As a side effect, the mean IBD probabilities in FS99 show that apparently slightly differing marker distances (M1 compared to M4) do affect the profiles of IBD parameters.

**Figure 3:**
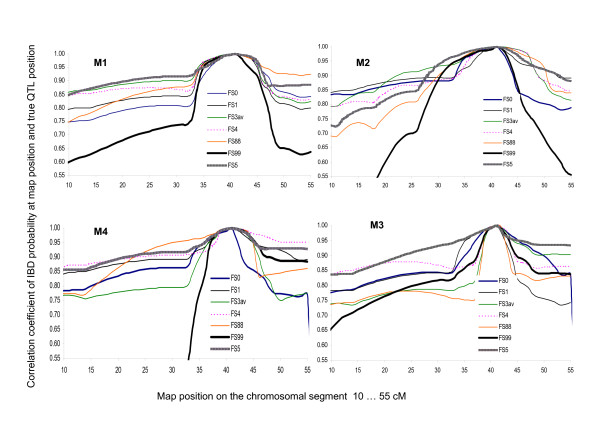
**Average profiles of Pearson's correlation coefficients of IBD at the current map position along the chromosomal segment (in cM on the x- axis) and IBD at the true QTL position (at 41.5 cM) for marker maps M1, M2, M3, M4 combined with single family structures**. FS0 (P < 0.0001 besides M4), FS1, FS3av (average of FS3, FS3a and FS3b), FS4 (P < 0.001 for M1), FS5 (P < 0.0001 for M1 and M3, P < 0.001 for M2), FS88 (P < 0.001 for M3, M4) and FS99 (P < 0.001 for M1, M4, P < 0.05 for M3).

Different profiles of correlations of IBD scores at the true QTL position and IBD scores along the map (profcorrIBD) could presumably be an indication of deviating reaction to simulation parameters, e.g. marker map (Figure 3). In general, correlations were smaller with increasing distance from the true QTL position. The reduction was stronger in combinations with more than two marker alleles (single plots not shown in detail). All family structures showed some variation in the steepness of profcorrIBD profiles (Figure 3). The overall message is clear: FS99, containing both the highest levels of inbreeding, IBD probability and relatedness, reached the steepest profcorrIBD in all combinations of simulation parameters. The profiles of FS99 became extremely steep, when the QTL- flanking markers were > 3.5 cM apart (as in M2 and M4). The reason for the large differences in profcorrIBD between FS99 and others is the same as for the profiles of average IBD probabilities and standard deviations (Figure 2). Parameters at the most distant map position at 0 cM and at the true QTL position (Table 1) were basically the frames for the steepness of correlation profiles profcorrIBD (Figure 3).

### QTL position estimates and pedigree relatedness

Except for FS5, the frequency of correctly estimated QTL positions (within an interval of 41.5 ± 1.5 cM on the marked chromosome) increased significantly with stepwise increasing inbreeding level (Figure 1). Most outliers resulted from analysing FS0. FS99 yielded the best results in terms of most correct QTL position estimates and least deviations from the actual QTL position.

Parallel runs via GridQTL mimicking a combined LD/LA- analysis yielded the same QTL position estimates as from the linkage analyses above, confirming robustness of QTL position estimates. When 100 sires and 100 dams were set up for a historic population 100 generations back, then the shape of the test statistic profile was similar to those obtained from the linkage analyses as described. The peak of the test statistic profiles became much sharper when choosing only two sires in the historic population (test statistic profiles not shown). This is another indication of the impact of a historically stronger related, and most likely more inbred, background when population history started with only two sires. In all cases, exactly the same situation in the pedigrees of FS0 to FS99 was given, whether 100 sires or two sires were chosen for a historical population. Hoffmann et al. (2000) stated that an older population with "reduced founder haplotypes by recombination" is more suited for fine mapping [13]. Subsequent generations of inbreeding as in FS99 could be advantageous in this sense as well. Thus, our results support the conclusions from analysing human pedigrees.

### Approaching the optimum inbreeding level

**Figure 4:**
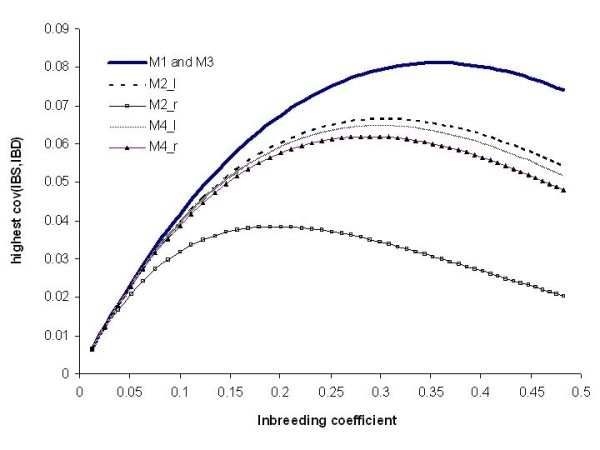
**Plot of covariance of IBD and IBS (cov(IBS, IBD)) against inbreeding coefficient based recombination rate (*c*) of nearest adjacent marker and QTL**. For marker maps M1and M3 recombination rates are equal (c = 0.01 of QTL and each bracketing marker), for M2 (c = 0.017 for left marker and QTL M2_l, c = 0.048 for right marker and QTL M2_r) and for M4 (c = 0.018 for left marker and QTL M4_l and c = 0.02 for right marker and QTL M4_r).

The relationship between increasing inbreeding level F_x _and cov(IBS, IBD) is not linear (Figure 4). The optimum of F_x _depends on the recombination rate of QTL and the nearest informative marker, being the same in marker maps M1 und M3 (*c *= 0.01). Higher N reduces cov(IBS, IBD) and thereby it reduces the optimum inbreeding level. The lower the recombination rate, the higher F_x _at which the maximum of cov(IBS, IBD) can be reached (Figure 4). Table 3 shows the effect of effective population size, generation number and allelic frequency of a trait locus on maximum F_x _and cov(IBS, IBD). In most cases (i.e. with recombination rate *c *= 0.01 of adjacent marker and QTL), the optimum in terms of maximum cov(IBS, IBD) was reached at F_x _= 0.35. Our family structures, except FS99 with F_x _= 0.28, were still far from reaching this optimum. An inbreeding level as great as in FS99 is not assumed to be relevant for today's dairy practice. But there is an old example (sire "Beltsville") serving as a proof for a very high level of inbreeding.

**Table 3:** Inbreeding coefficient F_x _, allelic frequency (*m*), number of generations (*t*) at maximum cov(IBS, IBD) for various effective population sizes (*N*) and for recombination rate *c *= 0.01 of QTL and adjacent marker

*N*	*t*	*m*	F_x_	cov(IBS, IBD)
10	12	0.5	0.45	0.105
		0.1	0.45	0.038
15	17	0.5	0.43	0.100
		0.1	0.43	0.036
20	21	0.5	0.41	0.096
		0.1	0.41	0.034
50	37	0.5	0.31	0.074
		0.1	0.31	0.026

### Outlook

Our results are not considered to encourage inbreeding for practical breeding. Inbreeding depression effects have to be avoided. But, a capacity for exploiting inbreeding for QTL study designs is still available [14]. However, as in each successful QTL analysis, the prerequisite is a QTL actually segregating in the pedigree to be studied. It should be mentioned, that estimation of breeding values, based on a relationship matrix incorporating pedigree information and genomic information, is still a topic in the literature, even with respect to the advanced dense SNP technology for genomic selection [[15] and [16]].

In this study, cov(IBS, IBD) is based on one marker only. We used the recombination rate of the nearest informative marker and QTL for calculating cov(IBS, IBD). The focus was on a practical pedigree as we can find in conventional dairy cattle breeding. The method can be extended to multiple markers. Further, a more general conclusion could be drawn by simulating pedigrees with random mating of diploid organisms with discrete generations and stepwise evaluating QTL estimates. Using a defined population history, such design could reveal an even higher average level of inbreeding (co-ancestry) than assumed with the two founder sires in our study.

## Conclusions

Our simulation study carried out with respect to realistic conditions in dairy cattle revealed intrinsic relationships between precision of estimated QTL positions and pedigree relatedness in the mapping population. IBD parameters obtained from analysing family structures with varying inbreeding load yielded conclusive results with respect to the meaning of inbreeding for QTL estimation and its dependence on relatedness. Related pedigrees are necessary for linkage analyses, and the stronger the relatedness is, the greater is the success of such studies. Comparing two versions of historic populations used in a GridQTL analysis that mimics a combined LD/LA- analysis additionally underlined the advantage of inbreeding and increased relatedness. This leads us to the assumption that linkage disequilibrium of markers and QTL across several generations could easier be detected than in non inbred or "less related" pedigrees. It must be noted that the relationship of the capacity for QTL detection (here, expressed by cov(IBS, IBD)) and the average inbreeding level of a population is not linear. Finally, these results apply to the situation of one biallelic QTL actually segregating in the pedigree, marked by a defined chromosomal segment.

## Methods

### Data simulation

**Figure 5:**
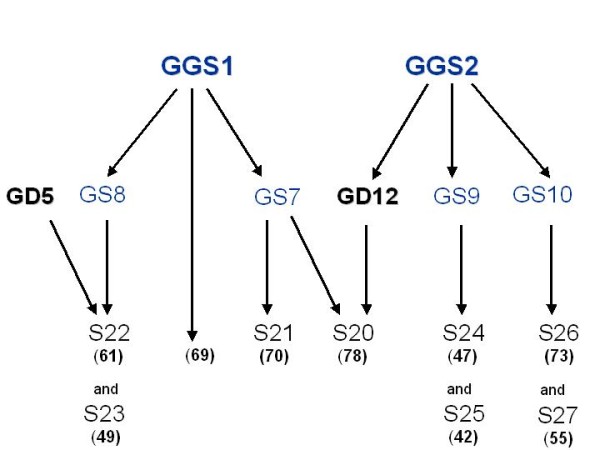
**Basic non-inbred family design FS0 with great grandsires (GGS1 and GGS2), grandsires (GS7, GS8, GS9, GS10), sires (S20, S21, S22, S23, S24, S25, S26, S27), number of final offspring per sire (in brackets), and grand dams (GD5 and GD12)**. GD5 and GD12 were the basis for the differences in inbred family structures.

The basis of the study design was a general pedigree structure comprising four generations with 850 individuals (Figure 5). GGS1 and GGS2 were male founders, followed by four grandsires and nine sires, with 42 to 78 offspring each (544 final offspring in total). Overlapping generations were also included in that one great grandsire, GGS1, was the sire of 69 final offspring. Both male founders were called 'great grandsires', regardless of overlapping generations.

Nine family structures (FS) were created with different inbreeding levels (described in detail in Table 4). FS0 included no inbreeding, as no parents were related (Figure 5). All other family structures consisted of the same numbers of generations and individuals as in FS0, but containing inbred mates. The inbreeding level increased from mild inbreeding in FS1 (F_x _= 0.0625 of one single sire) up to a higher level in FS88 (F_x _= 0.15 on average of all sires), and up to an extremely high inbred FS99 (F_x _= 0.28 in total, Table 4). Maternal structure in final offspring remained the same in all family structures (as in [8], Table 5). As the parents of four founder individuals (two great grandsires and two great grand-dams) and most of the female mates of the grandsires were unknown, their genotypes were sampled according to allelic frequencies. Their distribution was modeled to be in Hardy Weinberg equilibrium (approach as in Schelling et al., 1998) [17]. Specifically, diploid offspring genotypes composed by a maternal and a paternal gamete were assumed in Mendelian inheritance. Marker information and phenotypes of both great grandsires were equal in all family structures. Trait values (phenotypes) were generated as follows: Individual trait observation *y_i _*is based on a normally distributed QTL effect *q*, polygenic effect *g *(here, as a sum of 20 single gene effects) plus residual effect *e*, according to model

**Table 4:** Characteristics of the inbred family structures (FS), average inbreeding coefficients (F_x_) of sires of final offspring (SOF) and of the whole pedigree (F_x _total)

		F_x_	F_x_
FS	main characteristics	SOF	total
FS1	S20 originated from an aunt-nephew-mating with F_x _= 0.0625	0.007	< 0.01
FS3	S20 originated from mating half-sibs with F_x _= 0.125	0.014	< 0.01
FS3a	based on the same structure as FS3, but it contained three inbred sires, F_x _= 0.125 each, and offspring number of one sire (S20) in- creased to 138, while simultaneously reducing offspring number of GSS1 by 60	0.042	< 0.01
FS3b	based on the same structure as FS3, but the offspring number of S20 increased to 138, while simultaneously reducing final off- spring number of GGS1 by 60	0.014	< 0.01
FS4	extension of FS3 by one additional strongly inbred sire from a full- sib mating, where one sib was already inbred, F_x _= 0.375	0.056	< 0.01
FS5	contained two sires originating from a mother-son mating with F_x _= 0.375, and a sire from a half sib mating F_x _= 0.125, pedigrees of GGS1 and GGS2 remained fully separated from each other (this missing link was a remarkable deviation from all other FS)	0.097	< 0.01
FS88	contains all sires inbred, F_x _ranges from 0.063 to 0.375	0.150	0.01
FS99	an extremely inbred design already starting with inbred grand sires, F_x _of sires ranges from 0.250 to 0.426	0.290	0.28

**Table 5:** Overview of simulation parameters and symbols as used in the manuscript

Sets of simulation parameters in detail (code in brackets)
Four different sets of marker map:
Marker positions were given by marker maps in four versions (M1, M2, M3, M4), where marker position slightly varied (in marker distances) on the 55 cM long chromosomal segment
(M1) markers at 0, 13.7, 32.8, 35.7, **40.5, 42.5**, 43.5, 44.5, 45.5, 48.5 and 53.2 cM
(M2) markers at 0, 10.9, 17.8, 25.9, 33,6 **39.8, 46.3**, 48.3, 49.3, 51.2 and 54.4 cM
(M3) markers at 0, 13.7, 32.8, 35.5, 37.5, 39.3, **40.3 42.4**, 43.3, 44.3 and 48.4 cM
(M4) markers at 0, 13.7, 32.8, 35.7, 37.7, **39.7, 43.5**, 44.5, 45.5, 46.5 and 49.4 cM
Bold script marks the marker bracket harbouring the QTL at 41.5 cM in each map

Marker information (three different sets regarding the number of marker alleles):
(2_A) two marker alleles
(4_A) four marker alleles
(6_A) and six marker alleles

Number of analyses (= repetitions per family structure):
**4 **versions of marker maps **by 3 **versions of marker allele numbers **by 5 **variations individual missing values (in marker information and/or phenotypic values, combinations of 20% randomly missing values each) = **60 **data sets per family structure (60 independent analyses per family structure, with all parameters equally distributed)

(1) $$y_{i}=q_{i}+\sum_{20}g_{i}+e_{i}.$$

QTL effect was assumed to be normally distributed with mean zero and QTL- variance *σ_q_*^2^, contributing 15% of the total trait variance, based on additive and dominance effects of the QTL (*a *and *d*) and allelic frequencies (*m *and *n*) [18],

(2) $$\sigma_{q}^{2}=m^{2}{\left[2n(a-md)\right]}^{2}+2mn{\left[a(m-n)+d(1-2nm)\right]}^{2}+m^{2}{\left[-n(a+md)\right]}^{2}.$$

The polygenic component, contributing 25% of the total variance, was assumed normally distributed with mean zero and additive polygenetic variance, of single genes (*g*) with small effects of alleles *l *and *k *at each locus,

(3) $$\sigma_{g}^{2}={\left[2mnl(1+k(m-n))\right]}^{2}.$$

A random deviate *e *was normally distributed with mean zero and variance σ_res_^2 ^(i.e. 60 percent of the total variance). Recombination events were simulated on the basis of a binomial map function. Trait values and marker genotypes were simulated in an identical manner for all family structures, applying the PEDSIM approach [17].

Sixty datasets were simulated for each family structure, based on variations in three simulation parameters (Table 5): (i) marker positions on the chromosomal segment (according to marker maps M1, M2, M3 and M4), (ii) marker allele numbers (2 marker alleles, 4 marker alleles and 6 marker alleles), and (iii) five versions of individual information content in terms of missing value distribution. The 11 micro satellite markers were unevenly distributed as in a situation of real QTL- mapping, preceding the fast developing SNP technology and genomic selection. The pre-fine-mapping QTL study covered a 55 cM chromosomal segment, which was expected to harbor one QTL. The focus of this study was clearly on detecting inbreeding effects on parameters of the IBD probability in consideration with estimated QTL map position. Thus, principal conclusions on them do not depend on the kind of molecular markers, numbers of markers or marker alleles. The 60 data sets per family structure were repetitions. Using this term is comparable to successive health data collections, in the same patients and their families, at different times of life, in different clinics, or treatments simultaneously affecting all families at a time in the same way. Statistical parameters were calculated by using SAS package, version 9.1 (SAS Institute, Inc., Cary, NC), and effects of simulation parameters on IBD scores were tested with proc GLM.

### Calculating IBD probabilities and QTL analysis

The QTL effect was assumed random, with co-variance structure between individuals being a function of IBD probabilities at a particular location. A Fortran 90 program for calculating IBD probabilities was written that enables exploiting as much available information on pedigrees and markers as possible [19]. The kernel of the program package was the rapid deterministic recursive algorithm for calculating IBD probabilities between each pair of gametes [5], followed by transmission of marker alleles from parents to offspring [2]. Further, a method by Knott and Haley (1998) was implemented to determine IBD probabilities among (full) sibs' gametes in the second generation [7].

IBD probabilities were calculated for each pair of gametes independently, to obtain a matrix **G_p _**of gametic IBD probabilities at each position (p) in the chromosomal segment. Then, a mixed model was applied

(4) $$y=X\beta +Zu+H_{p}a_{p}+e,$$

where **y **is an (*n_1 _*× 1) vector of phenotypes. *n_1 _*refers to the number of animals with phenotypes, and *n_0 _*is the total number of animals in the pedigree. **X **is an (*n_1 _× *s) design matrix of a number of fixed effects (*s*), **Z **is an (*n_1 _× n_0_*) incidence matrix relating animals to their phenotypes, **H_p _**is a (*n_1 _× 2n_0_*) incidence matrix relating animals to paternal and maternal QTL alleles at position *p*, **β **is an (*s × 1*) vector of fixed effects, **u **is an (*n_0 _× 1*) vector of random polygenic effects, **a_p _**is an (*2n_0 _× 1*) vector of the effect of a QTL at position p, and **e **is an (*n_1 _× 1*) residual vector with expectation and covariance matrix (0, **E **⊗ **I**), where **E **is the unknown (co-) variance matrix of the residual effects and **I **denotes the identity matrix. **X **is equal to **1**, since all phenotypes were assumed pre-adjusted for non-genetic effects, and thus s = 1 and β = *μ*. The random polygenic effects **u**, and QTL effect **a_p_**, are assumed to follow a normal distribution with mean zero and variances **A*σ***^2^*_u _*and **G_p_***σ*^2^*_p_*, respectively. Matrix **A **is the additive relationship matrix. Matrix **G_p _**contains IBD probabilities at position *p*, obtained as described above. The model was fitted for each single position p (in steps of 1 cM) on the chromosomal segment. The data were analyzed using a random model variance component approach. The residual maximum likelihood (**REML**) procedure implemented in the ASReml software [3] was used to maximize the likelihood under both H_0 _and H_A _given the parameters for computing the likelihood ratio **LR **= -2(lnL_H0_-lnL_HA_) ~*χ*^2^_df_, to be calculated at each position p to find the most likely QTL position, with lnL_H0 _the logarithm of the likelihood computed for the pure polygenic model, and ln L_HA _the logarithm of the likelihood from the QTL model.

### Parallel analyses assuming combined linkage disequilibrium and linkage

The QTL estimates obtained by linkage analysis (LA) as described above were compared by results of independent analyses using GridQTL. Thereby, the R- method that is based on a regression model was adapted [20]. The advantage of this method is that it only requires genotypes instead of haplotypes to establish the "historical generation" [21]. Here, 100 historical generations back to the defined pedigree design were chosen to mimic linkage disequilibrium (LD). Two extreme versions (two and hundred sires for mating to 100 cows each) characterized the effective population size of the historical generation. This step enabled analysing the data in terms of combined LD/LA [20,21].

### Covariance of IBS and IBD and inbreeding level

The IBD matrix at the hypothetical QTL position is constructed using the identity by state information (IBS) from nearby markers. Thus, the covariance between IBS at a marker and IBD at a QTL (cov(IBS, IBD)) is a good parameter to study the relationship between inbreeding and ease of QTL detection. This parameter was defined as

(5) $$\sigma (IBS_{M},IBD_{QTL})=\left(F_{M,QTL}-F_{M}F_{QTL}\right)\left(1-\Pi_{M}\right),$$

where *F*_*M, QTL *_is the joint IBD at the marker and the QTL, *F*_*M *_and *F*_*QTL *_are the IBD (inbreeding) at the marker and QTL, respectively [20]. Π_M _is the initial homozygosity at the marker (equivalent to *m*^2 ^for an allele with frequency *m*). For the founder population, we assume a randomly mating population that started with no inbreeding or co-ancestry, and *F_M_*=*F_QTL _*= 1-e^-t/2N^. If the recombination rate *c *between marker and QTL is small and the effective population size (*N*) is large, then (according to Hill and Hernandez-Sanchez 2007) [22]

(6) $$F_{M,QTL}=1-X_{M}-X_{QTL}+X_{M,QTL}$$

and

(7) $$X_{M,QTL}(t+1)\approx (1-2c-1/2N)X_{M,QTL}(t)+2cX_{M}(t)X_{QTL}(t),$$

where t denotes generation number and X non-ibd (i.e. F = 1-X). Here, the recombination rate c was taken for the nearest informative marker and the QTL, depending on the marker distances in each marker map (Table 5). We used cov(IBS, IBD) to determine the optimum level of inbreeding for QTL detection in the examined population.

## Authors' contributions

NV wrote the Fortran code for the IBD calculations and adapted it to the design. GF and NV worked on the study design and did the simulations and analyses. JHS contributed to the methods part and to discussing results. GF suggested the basic questions to be studied, suggested discussions and drafted the manuscript. All authors approved the final manuscript.
